# Complete mitochondrial genome of Red bigeye (*Priacanthus macracanthus*): genome characterization and phylogenetic analysis

**DOI:** 10.1080/23802359.2018.1491334

**Published:** 2018-07-11

**Authors:** Yuena Sun, Tianjun Xu

**Affiliations:** a Key Laboratory of Exploration and Utilization of Aquatic Genetic Resources (Shanghai Ocean University), Ministry of Education, China;; b International Research Center for Marine Biosciences at Shanghai Ocean University, Ministry of Science and Technology, China;; c Key Laboratory of Freshwater Aquatic Genetic Resources, Ministry of Agriculture, China

**Keywords:** Priacanthidae, *Priacanthus macracanthus*, mitochondrial genome

## Abstract

Red bigeye, *Priacanthus macracanthus,* a species in the family Priacanthidae was added to the seafood red list by Greenpeace International due to the decline in its abundance and overfishing. To understand the phylogenetic relationship of Red bigeye in teleost, we determined the complete mitochondrial genome of Red bigeye. The complete mitochondrial genome is 17,003bp in length, including 13 protein-coding genes, 2 ribosomal RNA genes, 22 transfer RNA genes, and 2 main non-coding regions. Phylogenetic tree shows that Red bigeye is clustered with the fishes of the order Perciformes.

Priacanthidae (bigeyes), is a family of 4 genus and 19 species of marine fishes. Being a member of the family Priacanthidae, the Red bigeye (*Priacanthus macracanthus*) is native to the tropical and subtropical parts of southern Japan to western Indonesia, the Arafura Sea, and Australia. To determine the taxonomic resolution and phylogenetic relationships of Red bigeye with other vertebrates, complete nucleotide sequence of mitochondrial genome of Red bigeye was sequenced in this study. The samples were collected from Zhoushan Islands in the East China Sea (29.9′N, 122.2′E); the specimen was kept at the Museum of Marine Biology at Zhejiang Ocean University.

The mitogenome of Red bigeye is a closed, double-stranded circular molecule of 17,003 nucleotides (GenBank accession number: NC_029222) and contains 13 protein-coding genes, 2 rRNA genes, 22 tRNA genes, the control region (CR), and the origin of the light strand replication (O_L_). The base composition is T 26.3%, C 30.0%, A 27.0%, and G 16.8%. The A+T (53.3%) content is higher than G+C (46.7%) content, which is similar to other fishes (Cheng et al. 2012; Jin et al. 2013). Furthermore, the anti-G bias is ascertained in the third position of the protein-coding genes, which brings Red bigeye in line with other vertebrate mitogenomes (Liu et al. 2013). The two ribosomal RNA genes 12S rRNA (956bp) and 16S rRNA (1698bp) are located on the heavy strand between tRNA^Phe^ and tRNA^Leu^ (UUR), and are separated by tRNA^Val^ gene. The 13 protein-coding genes are encoded on the heavy strand but ND6 is encoded on the light strand. All the protein-coding genes start with ATG, except for ND4 which uses GTG as the initiation codon. The stop codon of four protein-coding genes (ND1, ATP8, ND4L, and ND5) is TAA, while COI and ND6 end with TAG. The remaining protein-coding genes (ND2, COII, ATP6, COIII, ND3, ND4, and Cytb) have incomplete stop codon, which are either TA or T, common to vertebrate mitochondrial protein-coding genes (Xu et al. 2011). And these incomplete termination codons may complete as TAA via posttranscriptional polyadenylation (Ojala et al. 1981). The 22 tRNA genes, including two forms of tRNA^Ser^ (UCN and AGY) and tRNA^Leu^ (UUR and CUN), scatter throughout the genome and range from 67 to 74 bp in size, and the gene arrangement is typically similar to most of the vertebrates. Among the 22 tRNA genes, 13 are located on the heavy strand and 9 on the light strand. Three tRNA clusters (IQM, WANCY, and HSL) are well conserved in Red bigeye, same as in other vertebrate mitochondrial genomes (Wei et al. 2013). Some overlaps occur in protein-coding genes and tRNAs ranging from 1 to 10 bp, which is similar to most of the vertebrates. The control region that length, determined to be 1030bp which is located between tRNA^Pro^ and tRNA^Phe^ and this characterization is consistent with those of the other teleost. Remarkably, the conserved motif 5’-GCCGG-3’ was identified in the mitogenome of Red bigeye, which is involved in the transition from RNA to DNA synthesis. Phylogenetic analysis results that based on the complete mitochondrial genome sequences, demonstrated that Red bigeye was clustered with the fishes of the order Perciformes (Figure 1).

**Figure 1.**
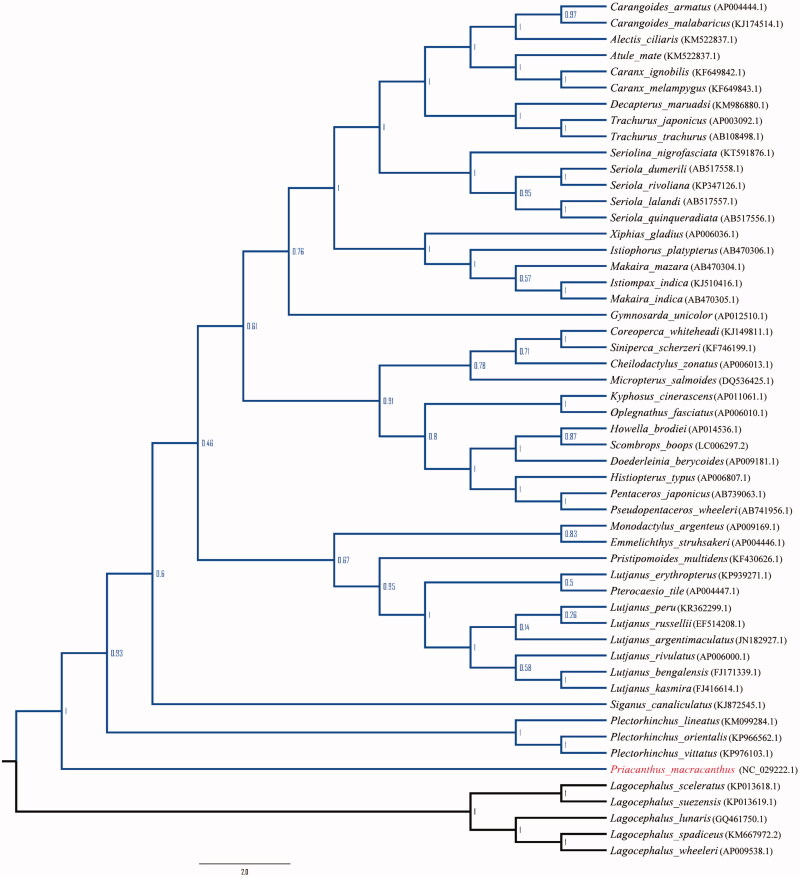
Phylogenetic tree was constructed using the neighbor-joining based on the complete mitochondrial genome sequences.
